# Second‐Harmonic Generation Enhancement in Polar Dielectric Films via Nonlocal Longitudinal Modes and Berreman Modes

**DOI:** 10.1002/nap2.70298

**Published:** 2026-09-18

**Authors:** Yining Yao, Ye Zhang, Jie Luo, Dongliang Gao, Hao Hu, Lei Gao

**Affiliations:** ^1^ School of Physical Science and Technology, Collaborative Innovation Center of Suzhou Nano Science and Technology, Jiangsu Key Laboratory of Frontier Material Physics and Devices Soochow University Suzhou China; ^2^ National Key Laboratory of Microwave Photonics, College of Electronic and Information Engineering Nanjing University of Aeronautics and Astronautics Nanjing China; ^3^ Jiangsu Key Laboratory of Biophotonics Suzhou Key Laboratory of Biophotonics SZCU‐ITU International Joint MetaCenter for Advanced Photonics and Electronics Suzhou City University Suzhou China

**Keywords:** Berreman mode, nonlocal effects, polar dielectrics, second‐harmonic generation

## Abstract

Polar dielectrics offer considerable promise for mid‐infrared nanophotonics and nonlinear optics because of their low optical losses and spectral bands of negative permittivity. However, previous studies have generally shown that nonlocal effects in polar dielectrics lead to suppression effects. This work reveals a different phenomenon, whereby we utilize the nonlocal effect to achieve second‐harmonic generation (SHG) enhancement in polar dielectric films. Specifically, the longitudinal‐mode resonances in polar dielectrics introduce multiple longitudinal resonances. We combine the nonlocal longitudinal mode at the fundamental frequency with the Berreman mode at the second‐harmonic frequency, achieving simultaneous resonance of the fundamental and second‐harmonic waves, thereby significantly enhancing the intensity of SHG. Numerical results show that the second‐harmonic intensity in the nonlocal case can be hundreds of times higher than that in the local case. Our study indicates that nonlocality‐enhanced SHG provides a promising platform for the application of polar dielectric crystals in nonlinear nanophotonics.

## Graphical abstract

Nonlocal longitudinal resonances in ultrathin AlN enable thickness‐controlled harmonic matching between a resonance at the fundamental frequency and the Berreman resonance at the second‐harmonic frequency. The resulting simultaneous field enhancement at both frequencies substantially amplifies the predicted second‐harmonic response, revealing a double‐resonance mechanism for enhancing nonlinear light–matter interactions in deeply subwavelength polar dielectric films.

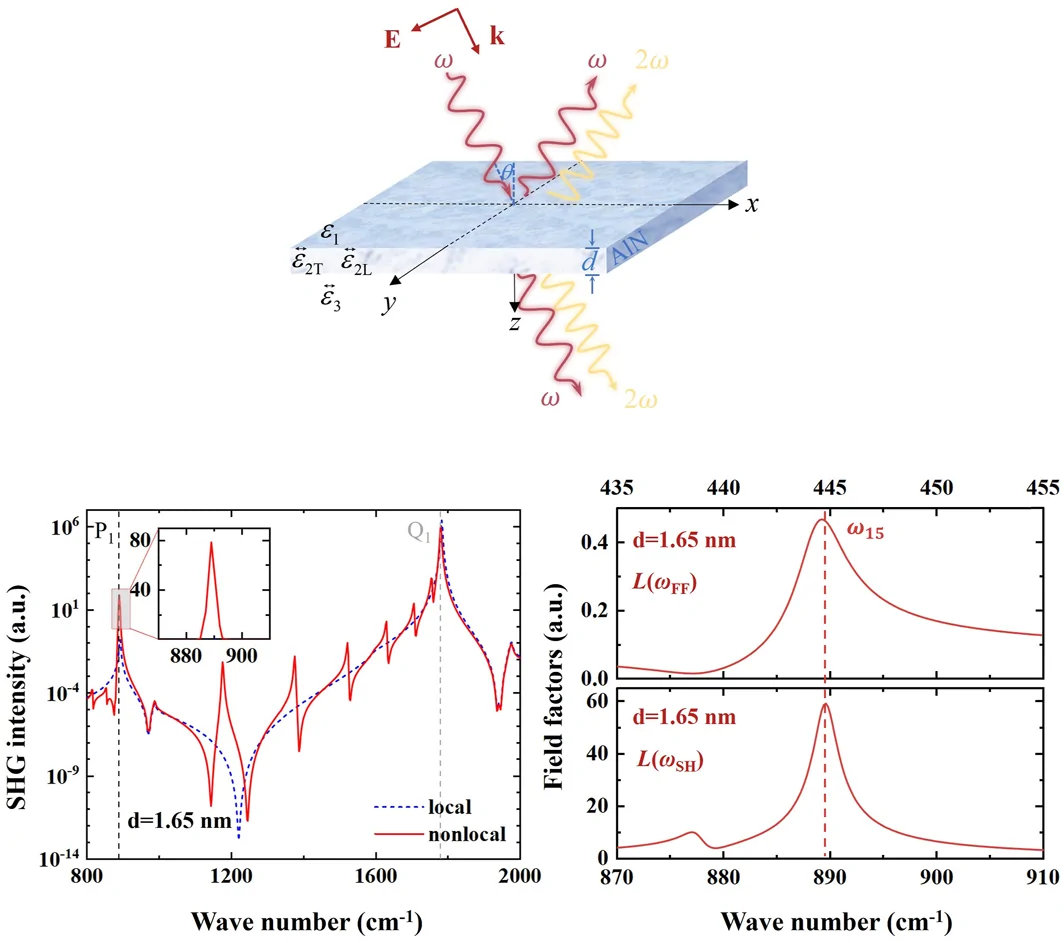

## 1 Introduction

Since the initial observation of second‐harmonic generation (SHG) in 1961, enhancing nonlinear optical processes has remained a core focus in optical research [1]. As one of the most fundamental nonlinear optical processes, SHG plays a major role in laser technology and optical communications [2, 3], biomedical imaging and sensing [4], materials science and surface characterization [5, 6, 7, 8], as well as quantum information and integrated photonics [9]. Numerous applications relying on SHG have been widely explored [10], and substantial research has focused on harmonic generation in nanostructures [11, 12]. In the field of nanophotonics, the short interaction length typically results in low nonlinear conversion efficiency, and conventional phase‐matching or quasi‐phase‐matching schemes are difficult to implement, which greatly restricts the conversion efficiency of SHG [13]. Therefore, specific configurations are required to provide electric field enhancement for boosting SHG, such as photonic crystals [14, 15] or microcavity resonances [16, 17, 18]. Similarly, the intense field enhancement in subwavelength regions on metallic nanostructure systems or rough metal surfaces induced by surface plasmon polariton resonances can also significantly strengthen SHG [19, 20]. Such strongly enhanced local fields have advanced SHG‐based nanoscale applications including all‐optical switching [10, 21] and highly efficient sensing [22]. Among various approaches, resonance enhancement is a highly effective technique for improving SHG efficiency [12, 23, 24]. It is essentially a cavity‐enhanced strategy, centered on achieving simultaneous resonance of both the pump light (fundamental frequency) and the generated signal light (second harmonic) through structures such as Fabry–Pérot cavities [17, 25], microring resonators [26], or metasurfaces [27]. Compared with single‐resonance schemes, where only the fundamental frequency is resonantly enhanced, the resonance enhancement mechanism exploits the field enhancement effects at both optical frequencies, thereby greatly improving the nonlinear conversion efficiency of SHG.

In recent years, a widely discussed direction in nanophotonics has been the polariton modes emerging in plasmonic or polar dielectric subwavelength thin films when the material operates in the epsilon‐near‐zero (ENZ) regime, and such materials have been extensively investigated in both linear [28, 29, 30] and nonlinear optics [31, 32, 33, 34]. High Ohmic losses in metals significantly restrict their use in high‐quality optical resonances and nonlinear enhancement. Polar dielectrics, owing to their long phonon lifetimes and low optical losses, are regarded as promising alternative optical materials, with broad application prospects in low‐loss, deep‐subwavelength mid‐infrared nanophotonic platforms [35]. Surface phonon polaritons (SPhPs) in polar dielectrics enable efficient light field confinement at subwavelength scales. Recently, significant progress has been made in the study of SHG from SPhPs, with a focus on effects such as the coupling between localized and propagating modes and critical coupling enhancement [36, 37]. By tuning the period of nanopillar arrays or the prism gap width, SHG signals can be efficiently excited and detected, revealing pronounced differences between linear reflectivity spectra and nonlinear responses in the mode hybridization region [38]. As the size of polar dielectric material systems shrinks into the nanoscale, the local dielectric theory originally used to describe such systems will no longer be applicable, and nonlocal effects emerge, which can suppress the local field intensity to a certain extent. Nonlocality can act as a new degree of freedom to modulate polariton coupling, inducing double resonance and long‐period oscillation behaviors [39], as well as longitudinal mode splitting and hybridization in polar dielectrics such as SiC and AlN [40], resulting in distinctive time delay and beat frequency oscillations in the time‐domain response [39]. However, previous studies on the enhancement of SHG in nonlocal systems have mainly focused on plasmonic materials. Existing investigations of SHG in polar dielectrics are mostly restricted to the local regime, whereas research on SHG in nonlocal polar dielectrics remains scarce.

In this work, we investigate the linear and nonlinear optical responses of an ultrathin hexagonal AlN polar layer (below 10 nm), either freestanding or supported by a SiC substrate, where the observed SHG arises from the second‐order nonlinear polarization of bulk AlN. By adjusting the thickness of the ultrathin polar layer, we demonstrate that when a nonlocal longitudinal resonance at the fundamental frequency is harmonically matched to the Berreman resonance, the local electric field is significantly enhanced, leading to greatly improved SHG efficiency.

## 2 Methods

The structure considered here is represented in Figure 1a, where an anisotropic polar dielectric film of hexagonal AlN with thickness d is illuminated by a transverse‐magnetic (TM) plane wave. The structure consists of three layers numbered 1, 2, and 3 from top to bottom. Layer 1 is a semi‐infinite air layer, layer 2 is a hexagonal AlN thin‐film layer serving as the intermediate layer, and layer 3 is a semi‐infinite substrate layer that can be either air or anisotropic SiC. In the superstrate, namely air, the electric and magnetic fields are as follows:

(1) $$H_{1}=\left(A_{1}e^{ik_{1z}z}+B_{1}e^{-ik_{1z}z}\right)e^{i\left(k_{x}x-\omega t\right)}e_{y},$$

(2) $$E_{1}=\left[\frac{k_{1z}}{\omega \varepsilon_{0}\varepsilon_{1}}\left(A_{1}e^{ik_{1z}z}-B_{1}e^{-ik_{1z}z}\right)e_{x}-\frac{k_{x}}{\omega \varepsilon_{0}\varepsilon_{1}}\left(A_{1}e^{ik_{1z}z}+B_{1}e^{-ik_{1z}z}\right)e_{z}\right]e^{i\left(k_{x}x-\omega t\right)},$$

where the out‐of‐plane wave vector $k_{1z}$ in the above equations satisfies the relation $k_{1z}^{2}=k_{0}^{2}\varepsilon_{1}-k_{x}^{2}$. In addition, $k_{0}=\omega /c$ is the wave vector in vacuum and $k_{x}=k_{0}\;\sin\;\theta$ is the in‐plane wave vector.

**FIGURE 1:**
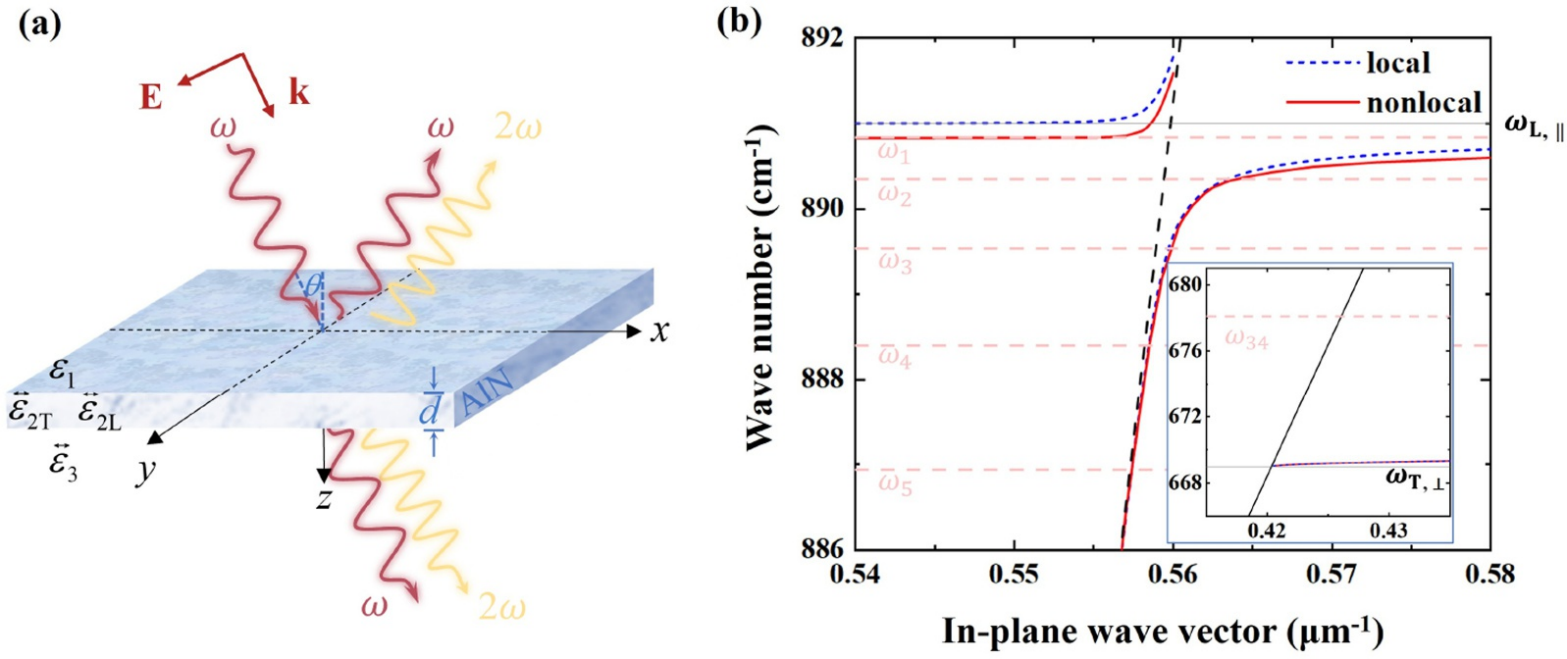
(a) Sketch of the polar thin film with thickness d illuminated by a TM wave. (b) Modal dispersion for a 5 nm AlN film. The red solid and blue dashed lines indicate the nonlocal and local dispersion. The black dashed line denotes the light line $k_{0}$ and the gray horizontal lines indicate $\omega_{L,∥}$ and $\omega_{T,⊥}$ of AlN (in the inset). The light red dashed lines indicate the nonlocal longitudinal modes.

Without loss of generality, we consider a Cartesian coordinate system with its *z*‐axis parallel to the crystal axis of the second layer. Accordingly, the transverse permittivity tensor of the hexagonal AlN can be written as follows:

(3) $${\overset{⃡}{\varepsilon }}_{2T}=\left(\begin{array}{ccc} \varepsilon_{2T,⊥} & 0 & 0\\ 0 & \varepsilon_{2T,⊥} & 0\\ 0 & 0 & \varepsilon_{2T,∥} \end{array}\right),$$

where the permittivity components have the form $\varepsilon_{2T,u}=\varepsilon_{2\infty ,u}\left[\omega_{2L,u}^{2}-\omega \left(\omega +i\gamma_{2,u}\right)\right]/\left[\omega_{2T,u}^{2}-\omega \left(\omega +i\gamma_{2,u}\right)\right]$ (u=⊥,∥). Here $\varepsilon_{2\infty ,u}$ is the high‐frequency permittivity, $\omega_{2T,u}$ and $\omega_{2L,u}$ are the transverse optical (TO) and longitudinal optical (LO) phonon frequencies of hexagonal AlN, respectively, and $\gamma_{2,u}$ is the damping constant. In addition, the subscripts ⊥ and ∥ represent the crystal directions perpendicular and parallel to the optical axis of the crystal. The field components within the polar layer satisfy the following equations:

(4) $$H_{2}^{T}=\left(A_{T}e^{ik_{2Tz}z}+B_{T}e^{-ik_{2Tz}z}\right)e^{i\left(k_{x}x-\omega t\right)}e_{y},$$

(5) $$E_{2}^{T}=\left[\frac{k_{2Tz}}{\omega \varepsilon_{0}\varepsilon_{2T,⊥}}\left(A_{T}e^{ik_{2Tz}z}-B_{T}e^{-ik_{2Tz}z}\right)e_{x}-\frac{k_{x}}{\omega \varepsilon_{0}\varepsilon_{2T,∥}}\left(A_{T}e^{ik_{2Tz}z}+B_{T}e^{-ik_{2Tz}z}\right)e_{z}\right]e^{i\left(k_{x}x-\omega t\right)},$$

where $k_{2Tz}^{2}=k_{0}^{2}\varepsilon_{2T,⊥}-k_{x}^{2}\varepsilon_{2T,⊥}/\varepsilon_{2T,∥}$ is the out‐of‐plane wave vector.

When the film thickness d is on the nanoscale, the nonlocality must be taken into account. The longitudinal permittivity tensor ${\overset{⃡}{\varepsilon }}_{2L}$ has the same form as Equation (3), but its components are given by $\varepsilon_{2L,u}=\varepsilon_{2\infty ,u}\left[\omega_{2L,u}^{2}-\omega \left(\omega +i\gamma_{2,u}\right)-\beta_{2L,u}^{2}k^{2}\right]/\left[\omega_{2T,u}^{2}-\omega \left(\omega +i\gamma_{2,u}\right)-\beta_{2L,u}^{2}k^{2}\right]$, where $\beta_{2L,u}$ is a material‐dependent phenomenological velocity scale representing the effective mechanical coupling between longitudinal ionic displacements in neighboring unit cells. It determines the curvature of the LO phonon dispersion and hence the strength of spatial dispersion. A larger $\beta_{2L,u}$ produces more pronounced nonlocal corrections and a longer characteristic length scale of the longitudinal lattice response [40]. In this connection, the excitation of longitudinal waves within the hexagonal AlN nanolayer can be written as follows:

(6) $$E_{2}^{L}=\left[\frac{1}{\omega \varepsilon_{0}}\left(A_{L}e^{ik_{2Lz}z}+B_{L}e^{-ik_{2Lz}z}\right)e_{x}+\frac{k_{2Lz}}{\omega \varepsilon_{0}k_{x}}\left(A_{L}e^{ik_{2Lz}z}-B_{L}e^{-ik_{2Lz}z}\right)e_{z}\right]e^{i\left(k_{x}x-\omega t\right)}.$$

When the longitudinal permittivity is described in tensor form, the longitudinal wave vector can be determined by imposing the divergence‐free condition on the longitudinal electric displacement field, yielding $k_{x}^{2}\varepsilon_{2L,⊥}\left(\omega ,k_{x},k_{2Lz}\right)+k_{2Lz}^{2}\varepsilon_{2L,∥}\left(\omega ,k_{x},k_{2Lz}\right)=0$. Here we would like to mention that the in‐plane wave vector $k_{x}$ is dictated by the incident photonic field and conserved throughout the entire layered structure. Its magnitude is negligible relative to the lattice wave vector. Accordingly, only the unconstrained out‐of‐plane wave vector component $k_{2Lz}$ contributes to the nonlocal response [41]. Hence, only the out‐of‐plane (along the optical axis) longitudinal phonon velocity is retained and abbreviated as $\beta_{L}\equiv \beta_{2L,∥}$ , and the out‐of‐plane wave vector of the longitudinal electric field can be approximated as follows $k_{2Lz}^{2}=\left[\omega_{2L,∥}^{2}-\omega (\omega +i\gamma_{2,∥})\right]/\beta_{L}^{2}$. Because the wave propagates mainly along the crystal optical axis (the *z* direction), transverse lattice vibrations lie primarily in the direction perpendicular to the optical axis and correspond to $\omega_{T,⊥}$, whereas longitudinal lattice vibrations are mainly oriented along the optical axis and correspond to $\omega_{L,∥}$.

For the anisotropic semi‐infinite polar dielectric SiC substrate, the nonlocal effect is sufficiently weak to be reasonably neglected. Accordingly, a local anisotropic permittivity tensor is adopted to characterize the dielectric response of the substrate. This tensor shares an analogous form with the one given in Equation (3), as it is also described by the transverse Lorentzian permittivity. The electromagnetic fields in the substrate can be written as follows:

(7) $$H_{3}=\left(A_{3}e^{ik_{3z}\left(z-d\right)}+B_{3}e^{-ik_{3z}\left(z-d\right)}\right)e^{i\left(k_{x}x-\omega t\right)}e_{y},$$

(8) $${Ε}_{3}=\left[\frac{k_{3z}}{\omega \varepsilon_{0}\varepsilon_{3,⊥}}\left(A_{3}e^{ik_{3z}\left(z-d\right)}-B_{3}e^{-ik_{3z}\left(z-d\right)}\right)e_{x}-\frac{k_{x}}{\omega \varepsilon_{0}\varepsilon_{3,∥}}\left(A_{3}e^{ik_{3z}\left(z-d\right)}+B_{3}e^{-ik_{3z}\left(z-d\right)}\right)e_{z}\right]e^{i\left(k_{x}x-\omega t\right)},$$

where the out‐of‐plane wave vector is $k_{3z}^{2}=k_{0}^{2}\varepsilon_{3,⊥}-k_{x}^{2}\varepsilon_{3,⊥}/\varepsilon_{3,∥}$.

By simultaneously imposing the continuity conditions of electric and magnetic fields at both boundaries and further introducing the additional boundary condition of vanishing polarization component perpendicular to the polar dielectric interface in nonlocal theory, the nonlocal dispersion relation can be written as follows [42]:

(9) $$\left|\begin{array}{cccccc} 1 & -1 & -1 & 0 & 0 & 0\\ \frac{k_{1z}}{\varepsilon_{1}} & \frac{k_{2Tz}}{\varepsilon_{2T,⊥}} & -\frac{k_{2Tz}}{\varepsilon_{2T,⊥}} & 1 & 1 & 0\\ 0 & \Omega & \Omega & -1 & 1 & 0\\ 0 & e^{ik_{2Tz}d} & e^{-ik_{2Tz}d} & 0 & 0 & -1\\ 0 & \frac{k_{2Tz}}{\varepsilon_{2T,⊥}}e^{ik_{2Tz}d} & -\frac{k_{2Tz}}{\varepsilon_{2T,⊥}}e^{-ik_{2Tz}d} & e^{ik_{2Lz}d} & e^{-ik_{2Lz}d} & -\frac{k_{3z}}{\varepsilon_{3,⊥}}\\ 0 & \Omega e^{ik_{2Tz}d} & \Omega e^{-ik_{2Tz}d} & -e^{ik_{2Lz}d} & e^{-ik_{2Lz}d} & 0 \end{array}\right|=0,$$

where $\Omega =k_{x}^{2}/k_{2Lz}\;\left(1/\varepsilon_{2T,∥}-1/\varepsilon_{2\infty ,∥}\right)$. If nonlocality is neglected, that is, $\beta_{L}$ tends to 0, then $k_{2Lz}$ tends to ∞ and Ω tends to 0. Thus, the above equation reduces to the local case, which is $1+\left(\varepsilon_{1,}k_{3z}\right)/\left(\varepsilon_{3,⊥}k_{1z}\right)=i\;\tan\left(k_{2Tz}d\right)\left[\left(\varepsilon_{2T,⊥}k_{3z}\right)/\left(\varepsilon_{3,⊥}k_{2Tz}\right)+\left(\varepsilon_{1,}k_{2Tz}\right)/\left(\varepsilon_{2T,⊥}k_{1z}\right)\right]$.

With Equation (9), we can investigate how nonlocality modifies the dispersion relation. Furthermore, we can proceed to derive the reflectance and transmittance spectra, which enables a systematic analysis of its influence on optical absorption and even SHG.

## 3 Results and Discussion

To explore the influence of nonlocality on the optical response of the system, a brief analysis of the dispersion curves for an ultrathin film (d=5 nm) in air ($\varepsilon_{1}=\varepsilon_{3,⊥}=\varepsilon_{3,∥}=1$) is presented in Figure 1b. Note that Equation (9) is a transcendental equation with complex independent variables. To clarify the origin of the Berreman mode, we fix the real in‐plane wave vector $k_{x}$ and solve Equation (9) for the complex frequency. This approach is consistent with previous theoretical methods and is suitable for determining the complex eigenfrequency of the Berreman mode [37, 43].

Here, the high‐frequency permittivity components of hexagonal AlN used in our work are $\varepsilon_{2\infty ,⊥}=4.16$ and $\varepsilon_{2\infty ,∥}=4.35$; the TO phonon frequencies and LO phonon frequencies are $\omega_{2T,⊥}=669$ cm^−1^, $\omega_{2T,∥}=610$ cm^−1^, $\omega_{2L,⊥}=912$ cm^−1^ and $\omega_{2L,∥}=891$ cm^−1^; the damping constant is $\gamma_{2,⊥}^{\text{AlN}}=\gamma_{2,∥}^{\text{AlN}}=2.2$ cm^−1^ [37, 44]. The nonlocal parameter is $\beta_{L}=5.1\times 10^{5}$ cm s^−1^, which was deduced by fitting the measured reflectance spectra of superlattices [40, 45]. Figure 1b presents the local and nonlocal dispersion curves. In the local description, the two film branches occur at $k_{x}>k_{0}$, where the electromagnetic fields decay away from the layer. Their frequencies remain between $\omega_{T,⊥}$ and $\omega_{L,∥}$ of AlN. In the deeply subwavelength limit, the low‐ and high‐frequency branches (in the inset and in the main panel) asymptotically approach $\omega_{T,⊥}$ and $\omega_{L,∥}$, respectively. Over the considered range of $k_{x}$, the upper branch remains nearly pinned to $\omega_{L,∥}$, resulting in a nearly dispersionless response. Because Re(ε) is close to zero in this spectral region, this branch can be identified as an ENZ thin‐film polariton. The dispersion also extends into the radiative region $k_{x}<k_{0}$, where it forms the Berreman branch. Its frequency is nearly independent of $k_{x}$, apart from a small upward shift near the light‐line boundary. When nonlocal effects are introduced, the upper branch dispersion curve at $k_{x}>k_{0}$ rises synchronously with the increase of $k_{x}$ at small wave numbers, consistent with that in the local case. However, the nonlocal branch gradually deviates from and lies below the local branch at large in‐plane wave vector $k_{x}$. Consequently, the dispersion curve predicted by nonlocal theory lies below that of local theory. When $k_{x}<k_{0}$, the Berreman mode in the radiative regime under nonlocal conditions appears at a frequency slightly lower than the LO frequency and close to $\omega_{1}$. The thin films investigated in this work serve as Fabry–Pérot resonators for LO phonons, sustaining a set of discrete modes whose out‐of‐plane wave vectors are quantized and follow the relation $k_{2Lz}=n\pi /d$. The frequencies of the additional longitudinal modes (the light red dashed line) induced by nonlocality can be calculated using the following formula [41]:

(10) $$\omega_{n}=\sqrt{{\left(\omega_{2L,∥}\right)}^{2}-{\left(\frac{n\pi \beta_{L}}{d}\right)}^{2}}.$$

The Berreman mode can only be excited by a nonzero out‐of‐plane electric field component [46]. Therefore, an obliquely incident TM wave is required to excite the corresponding absorption peak. Figure 2a shows the absorption spectra of a single‐layer AlN thin film at an incident angle of 84°. In the local case, the Berreman mode of the film gives rise to an absorption peak at $\omega_{L,∥}$ of AlN. However, the peak absorption under nonlocal conditions decreases and exhibits a slight redshift to $\omega_{1}$, as predicted by the dispersion diagram in Figure 1b. In addition, nonlocality also induces a series of small peaks below $\omega_{L,∥}$. These discrete frequencies are determined by Equation (10) and correspond to odd values of n [47]. Figure 2b presents the absorption spectra of the air/AlN/SiC structure, where the hexagonal SiC substrate is anisotropic [48]. It is observed that the absorption characteristics of the air/AlN/SiC structure near the Berreman mode are analogous to those of the single‐layer thin film. Furthermore, in Figure 2c,d, we present the color map of the absorption as a function of incident angle near the frequency of the Berreman mode for the single‐layer AlN and the air/AlN/SiC structures in the nonlocal case, respectively. The results show that the Berreman absorption occurs at large incident angles, so 84° is chosen here as the incident angle for both the single‐layer film and the structure with a substrate.

**FIGURE 2:**
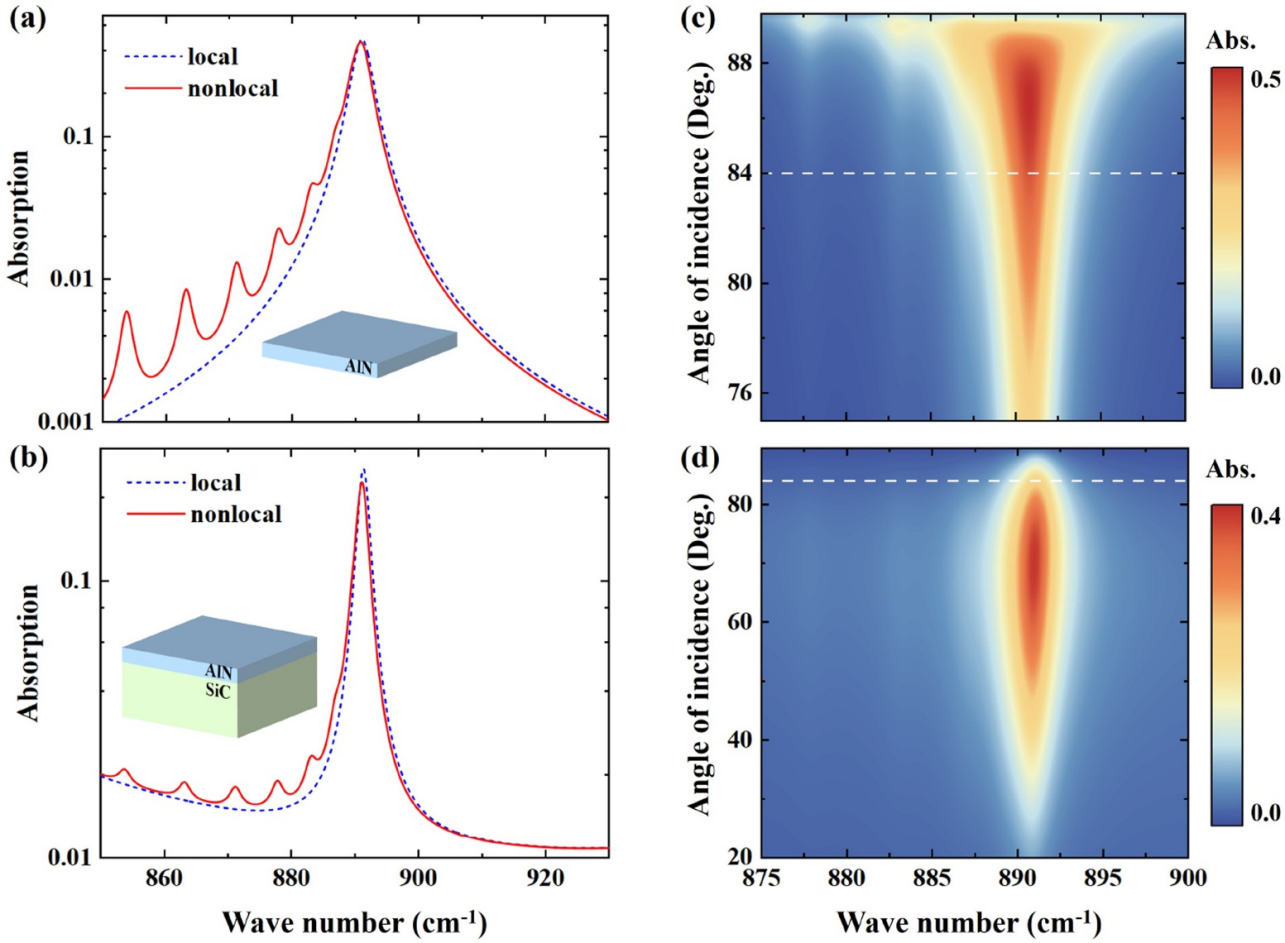
Absorption of (a) a freestanding AlN film with a thickness of 5 nm and (b) the structure with a substrate. The incident angle is 84°. The red solid and blue dashed lines indicate the nonlocal and local absorption. (c, d) Absorption near the frequency of the Berreman mode at different angles in the nonlocal case, corresponding to the structures shown on the left, respectively. The white dashed line denotes the location for an incident angle of 84°.

In this study, we attribute the dominant SHG process to hexagonal AlN, which possesses nonzero second‐order nonlinear susceptibility with independent components of $\chi_{\text{zzz}}^{\left(2\right)}$, $\chi_{\text{zxx}}^{\left(2\right)}=\chi_{\text{zyy}}^{\left(2\right)}$, $\chi_{\text{xxz}}^{\left(2\right)}=\chi_{\text{xzx}}^{\left(2\right)}=\chi_{\text{yyz}}^{\left(2\right)}=\chi_{\text{yzy}}^{\left(2\right)}$. In this process, the $\chi_{\text{zzz}}^{\left(2\right)}$ component exerts the strongest influence on SHG [49]. Meanwhile, for the structure investigated in this work, the amplitude of the out‐of‐plane electric field $E_{z}$ is much larger than that of the in‐plane electric field [37]. Therefore, we mainly discuss the effect of the $\chi_{\text{zzz}}^{\left(2\right)}$ component on the SHG process. The intensity of SHG scales with the square of the nonlinear polarization, expressed as follows [36, 50, 51]:

(11) $$I^{\text{SHG}}\propto \omega^{2}\eta^{\text{SHG}}\left(\omega \right),$$

where the enhancement factor is given by $\eta^{\text{SHG}}\left(\omega \right)={\left|L^{2}\left(\omega_{\text{FF}}\right)L\left(\omega_{\text{SH}}\right)\right|}^{2}$. Here, $L\left(\omega_{\text{FF}}\right)=E_{z}^{\text{FF}}/E_{z0}^{\text{FF}}$ stands for the spatially linear field amplification factor at the fundamental frequency $\omega_{\text{FF}}$, and $L\left(\omega_{\text{SH}}\right)=E_{z}^{\text{SH}}/E_{z0}^{\text{SH}}$ is the corresponding spatially nonlinear field amplification factor at the second‐harmonic frequency $\omega_{\text{SH}}$. This expression encapsulates the interaction between field amplifications at both frequencies in driving the nonlinear optical response.

Based on the above formalism, we compare the local and nonlocal models, choosing a moderate angle of 60° because the angle only influences the resonance strength, to investigate the distinctive optical characteristics and related physical effects of an ultrathin polar film with nonlocal response. As shown in Equation (10), these longitudinal modes are sensitive to both $\beta_{L}$ and thickness d. For the same material, $\beta_{L}$ is fixed, so here we discuss the effect of thickness. Figure 3a,b present the SHG intensity of a freestanding AlN film with d = 1.65 nm and d = 1.87 nm as functions of wave number at second‐harmonic frequency. In the local case, the SHG intensity exhibits enhanced peaks near two frequencies *P*_1,2_ and *Q*_1,2_. Near frequencies *P*_1,2_, the incident fundamental light itself does not undergo enhancement, whereas its generated second‐harmonic field due to second‐order nonlinear susceptibility can excite Berreman modes in the polar dielectric slab to achieve the enhancement of spatial fields, resulting in the enhanced peak. On the other hand, near frequencies *Q*_1,2_, the incident fundamental wave is enhanced via excitation of Berreman modes in the polar dielectric slab, whereas no additional enhancement mechanism exists at the second‐harmonic frequency. After the introduction of nonlocal effects, differences can be observed. In the nonlocal case, on the one hand, the SHG intensity at frequency *P*_1,2_ is significantly enhanced compared with that in the local case. This originates from the longitudinal mode resonance of the incident fundamental light wave under nonlocal conditions. Meanwhile, the second harmonic excites the Berreman mode in the thin film and thus achieves intensity enhancement. On the other hand, at frequencies *Q*_1,2_, the peak positions exhibit a slight redshift relative to the local model, and the nonlocal effect slightly suppresses the second‐harmonic intensity at these frequencies. In addition, below the LO frequency, a series of longitudinal modes can be excited, which gives rise to additional enhancements. As can be seen from the comparison of results for different thicknesses, the SHG intensity at frequency *P*_1,2_ is larger for thinner films (in the insets). In addition, Figure 3c,d display the SHG intensities of thin layers supported by a substrate, which exhibit a higher SHG intensity than the freestanding case.

**FIGURE 3:**
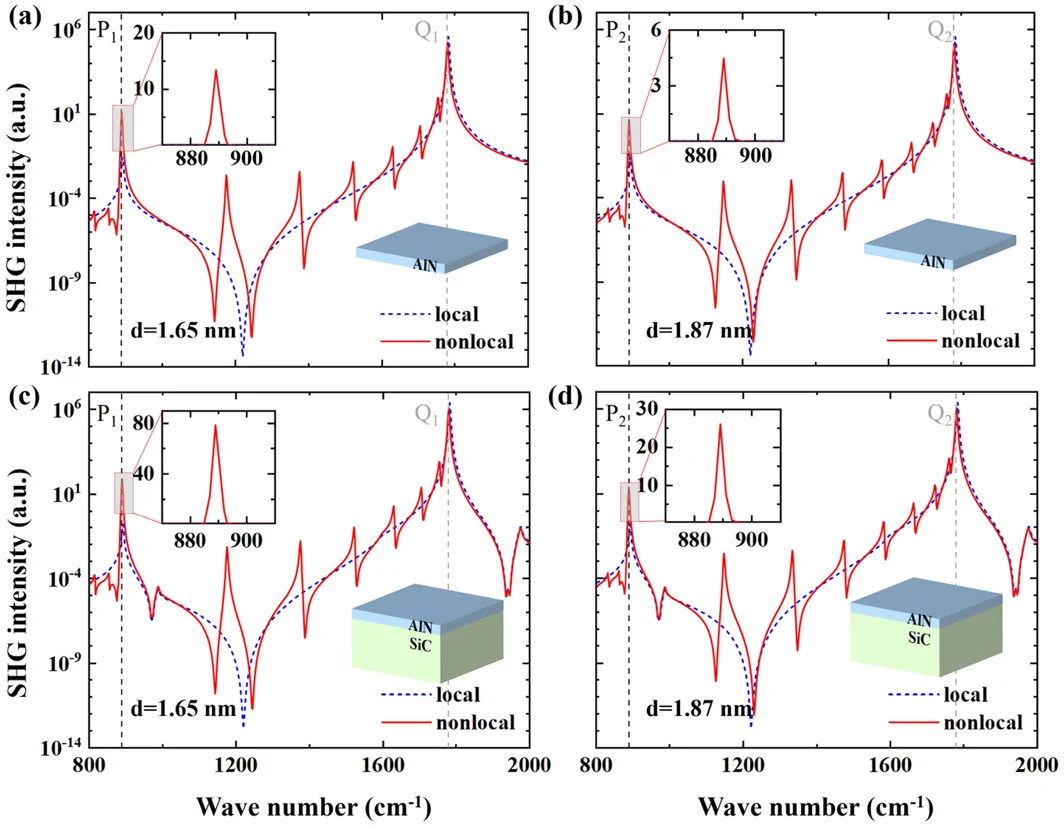
The local (the blue dashed lines) and nonlocal (the red solid lines) SHG intensities at the second‐harmonic frequency of single‐layer AlN with (a) d = 1.65 nm and (b) d = 1.87 nm. (c, d) Corresponding SHG intensities of AlN on a substrate at the second‐harmonic frequency. The incident angle is 60°. The frequencies marked by the black and gray dashed lines correspond, respectively, to the two frequency positions where the SHG intensity is significantly enhanced for each thickness under the nonlocal condition. The insets show enlarged views.

These longitudinal modes are highly sensitive to the thickness d (as shown in Equation (10)). Therefore, by adjusting the thin film thickness (such as the specific thicknesses of 1.65 and 1.87 nm adopted earlier), the fundamental wave excites the nonlocal longitudinal mode, whereas the generated second harmonic excites the Berreman mode. The simultaneous excitation of these two resonances produces the pronounced SHG enhancement. The coincidence of the two resonance peaks at this particular thickness leads to a dramatic enhancement of SHG intensity at frequency *P*_1,2_ in the nonlocal case, which is 2–3 orders of magnitude higher than that in the local case. This mechanism is quite different from metallic systems, in which nonlocal effects and the corresponding SHG enhancement can only be achieved above the plasma frequency [52, 53]. Moreover, surface plasmon polaritons in metals or heavily doped semiconductors suffer from intrinsic drawbacks such as short lifetimes and strong damping, whereas SPhPs in polar crystals possess much longer lifetimes owing to the persistent phonon resonances. The unique combination of low‐loss propagation and giant field amplification, both inherent to ENZ polaritons at LO phonon frequencies, makes these modes highly attractive for future nanophotonic device architectures.

To better clarify the physical mechanism behind the giant enhancement of SHG, we focus on the structure supported by a substrate, namely the air/AlN/SiC system. We present in Figure 4a,b the evolution of the fundamental field enhancement factor $L\left(\omega_{\text{FF}}\right)$ and the second‐harmonic field enhancement factor $L\left(\omega_{\text{SH}}\right)$ as functions of frequency near frequency *P*_1,2_ for the thicknesses of 1.65 and 1.87 nm, respectively. At these thicknesses, the resonant peaks of $L\left(\omega_{\text{FF}}\right)$ align with the resonant peaks of $L\left(\omega_{\text{SH}}\right)$. For these peaks of $L\left(\omega_{\text{FF}}\right)$, the parameter n in Equation (10) takes the values 15 and 17, respectively. The 15th and 17th small peaks of $L\left(\omega_{\text{FF}}\right)$ satisfy the relations $2\omega_{15}=\omega_{1}$ and $2\omega_{17}=\omega_{1}$. This accordingly leads to a pronounced enhancement of SHG intensity, as shown in the insets of Figure 3c,d. In fact, these two specific thicknesses can be analytically obtained from Equation (10). By varying the thickness d, multiple specific thicknesses satisfying $2\omega_{n}=\omega_{1}$ for the respective thickness (where n is an odd integer not equal to 1) can be found. In Figure 4c, we plot the SHG intensity as a function of second‐harmonic frequency and thickness, from which it can be observed that a giant enhancement of SHG intensity can be achieved at some specific thicknesses, where the resonant peak of $L\left(\omega_{\text{FF}}\right)$ is aligned with $L\left(\omega_{\text{SH}}\right)$. Figure 4d presents the maximum values of the SHG intensity for various thicknesses as discrete points (owing to the discrete thickness of AlN in actual preparation), which are mainly determined by whether $L\left(\omega_{\text{FF}}\right)$ and $L\left(\omega_{\text{SH}}\right)$ can be simultaneously enhanced. Therefore, by adjusting the layer thickness to satisfy the simultaneous resonance conditions, the SHG intensity can be greatly enhanced. From an experimental perspective, the fabrication techniques for ultrathin AlN are well established, and ultrathin AlN below 5 nm as well as its heterostructures have been extensively investigated [54]. Moreover, the actual material loss affects the resonance strength but does not necessarily change the double‐resonance mechanism.

**FIGURE 4:**
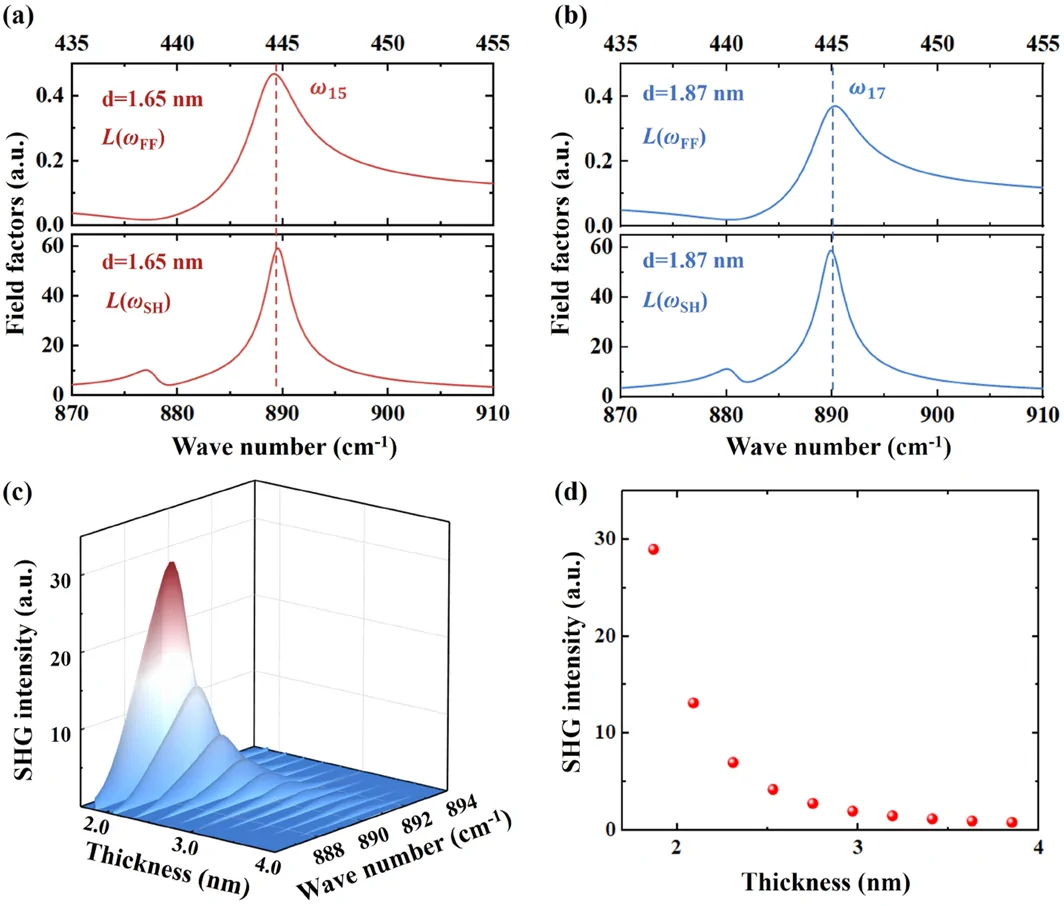
The mechanism of the SHG enhancement for nonlocal AlN on a substrate. Field enhancement factors at the fundamental and second‐harmonic frequencies for (a) d = 1.65 nm and (b) d = 1.87 nm. At these specific thicknesses, the frequency of the fundamental field resonance peak and that of the second‐harmonic field resonance peak satisfy a doubling relationship, and this position is marked by the dashed line. (c) The SHG enhancement factor at second‐harmonic frequency for various thicknesses. (d) The SHG intensity for different thicknesses.

For the layered structure in this work, the in‐plane electric field enhancement in the polar layer is negligible, whereas the out‐of‐plane electric field enhancement is dominant. In Figure 5, we present the out‐of‐plane electric field enhancement of the air/AlN/SiC structure under linear conditions, and $E_{z}$ is normalized to the incident field amplitude $E_{z0}$. Figure 5a shows the average electric field enhancement in the polar layer near the Berreman mode frequency for different thicknesses, and the white dashed lines indicate the maximum field enhancement for each thickness. It can be seen that the frequency corresponding to the maximum electric field changes with the thickness. Figure 5b plots the evolution of the out‐of‐plane electric field enhancement with frequency for different thicknesses. It can be seen that the average field enhancement exhibits a pronounced enhancement at the main peak $\omega_{1}$, accompanied by numerous additional small peaks to its left. At these two specific thicknesses, the 15th and 17th small peaks satisfy a doubling relationship with their respective main peaks. For clarity of demonstration, we calculate the electric field distribution in Figure 5c, where the thin film has a thickness of 1.65 nm. The figure clearly shows a strongly localized field enhancement within the polar layer at $\omega_{1}$. Meanwhile, a small peak occurs exactly at the frequency satisfying the frequency‐doubling relationship with the main peak. Here, the field is entirely confined within the polar layer, a fact mirrored by the smooth frequency response and modest amplitude of the $E_{z}$ field enhancement in the substrate. This strong field confinement and enhancement in the polar layer enable the enhancement of SHG.

**FIGURE 5:**
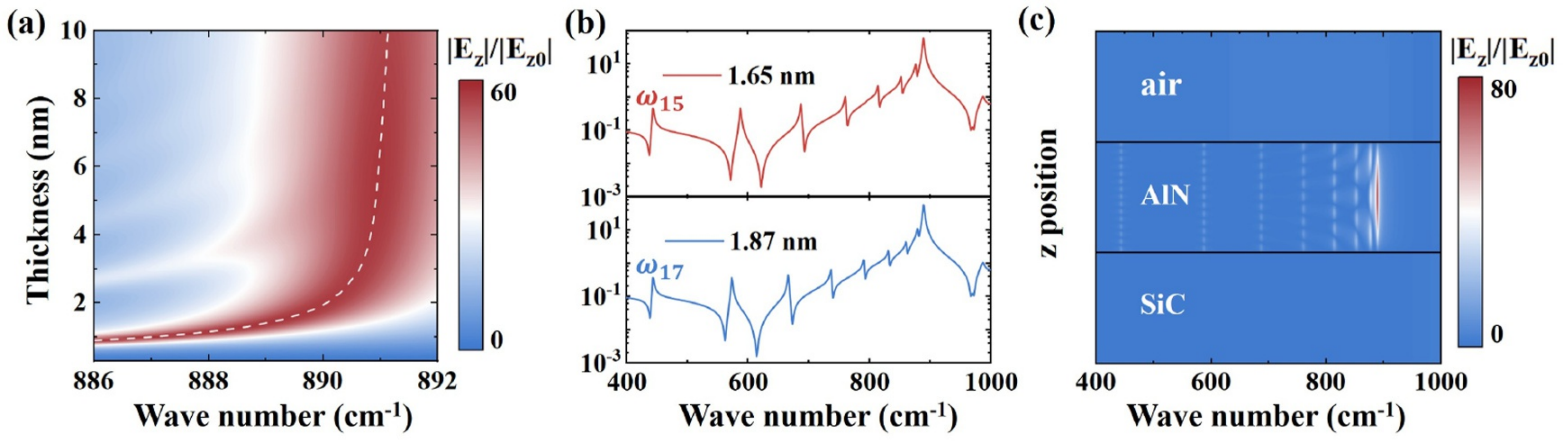
(a) The linear average out‐of‐plane electric field near the Berreman mode frequency for the nonlocal AlN layer at various thicknesses. The white dashed line traces the ridge of maximum field enhancement. (b) The linear average electric field for d = 1.65 nm and d = 1.87 nm. (c) The linear electric field distribution as a function of spatial coordinate *z* and frequency.

## 4 Conclusion

In summary, we employ the nonlocal response of polar dielectrics to realize SHG enhancement. We design a simple nanostructure that achieves resonant matching in the deep‐subwavelength regime, boosting the SHG intensity by two orders of magnitude. This enhanced SHG response arises from two factors. One is the additional resonances induced by nonlocal longitudinal modes. The other is the strong out‐of‐plane electric field amplification caused by the Berreman mode under second‐order optical nonlinearity, with the field confined within the ultrathin layer. The underlying mechanism lies in the overlap between the longitudinal mode resonances and the Berreman mode at the second‐harmonic frequency, which is achieved by tuning the thickness of the film. Given that SHG enhancement in local AlN has already been corroborated by previous experiments [37], we further predict that, when nonlocal effects are taken into account, the SHG spectra will evolve to exhibit multiple distinct resonance peaks. Crucially, the relevant nonlocal dispersion parameters can be quantitatively extracted by fitting the measured SHG data as a function of the AlN film thickness, providing a direct experimental route to verify our theoretical mechanism. This SHG enhancement strategy enabled by the nonlocal effect opens a new route for realizing efficient nonlinear optical control in low‐loss, deep‐subwavelength, and structurally simple platforms.

## Author Contributions

**Yining Yao:** conceptualization, methodology, software, validation, writing – original draft, visualization, investigation, data curation, writing – review and editing. **Ye Zhang:** writing – review and editing, supervision, software. **Jie Luo:** writing – review and editing, conceptualization, methodology. **Dongliang Gao:** writing – review and editing, funding acquisition. **Hao Hu:** software. **Lei Gao:** writing – review and editing, funding acquisition, conceptualization, methodology.

## Funding

The study was supported by the National Natural Science Foundation of China (12274314, 12574342); Suzhou Basic Research Project (SJC2023003); Jiangsu Key Laboratory of Biophotonics Open Projects Fund (2025BPT007).

## Conflicts of Interest

The authors declare no conflicts of interest.

## Data Availability

The data that support the findings of this study are available from the corresponding author upon reasonable request.
